# Annual Circulars of Medical Colleges

**Published:** 1850-09

**Authors:** 


### Part 4.—Editorial.

### ARTICLE I.

#### ANNUAL CIRCULARS OF MEDICAL COLLEGES.

The annual announcements for the next Course of Lectures of various medical schools are on our table.

In looking over these documents, one is forcibly struck with the uniform account of the great prosperity of each, and the superior advantages offered to students for acquiring a knowledge of medical science and the healing art. Nor do they generally fail to claim that the course of instruction given is thorough and *complete*. With this accumulated testimony from those who ought to know, what becomes of the complaints that have been ringing in our ears, from different parts of the country, in reference to the imperfections of medical schools? Certainly, if the pretensions of the colleges are just, the complaints are slanders.

But while the different schools are uniform in claiming to offer great facilities for instruction, and ability to teach, there are certain peculiarities in their pretensions, to which we would direct attention, by making a few extracts, which will give our readers some idea of medical college rhetoric.

In the "Report of the Medical Department of the University of Pennsylvania for the year 1850, by the Medical Faculty," we find the following language:—

"The vacancy thus created [by the resignation of Prof, Chapman], has with a wise regard to the interests of the school, been supplied by the appointment of Prof. Geo. B. Wood, the former eminent occupant of the chair of Materia Medica and Pharmacy. From the widely-spread reputation of Dr. Wood, as a medical writer and teacher; from the high sense, justly entertained, of his scientific attainments; and from his long connexion, as Prescribing Physician, with the

Pennsylvania Hospital, the Faculty have reason to be much pleased at his appointment; and feel well assured that his chair will be made signally effective. The Doctor is now on a visit to Europe, for the sole purpose of procuring such preparations, drawings, and other materials as may contribute to the value and usefulness of his lectures."

The announcement by the Trustees and Faculty of the Medical Department of the Iowa University, at Keokuk, a city of 3 or 4,000 inhabitants, gives the following.—

"In view of this arrangement [*i. e.*, an appropriation by the City Council for a hospital], the Department of Clinical Medicine has been added to the Chair of Pathology, filled since the organization of the College, by Professor Samuel G. Armor, whose abilities as a teacher are so extensively known. To those who have listened to Prof. Armor's able and interesting lectures on Special Pathology and Physical Diagnosis, nothing need be said of the wisdom of the cast. He will locate permanently in the city of Keokuk, and, aided by our eminent Professor of Theory and Practice, will make this department everything which the friends of Medical Science could wish."

The Annual Announcement of the Medical College of Ohio, begins an eulogy of nearly four pages on Professor Bell, as follows:—

"John Bell, M. D., of Phila., Professor elect of Theory and Practice, is well known, by reputation, to every physician in the United States; indeed, he is known as one of the most copious and able writers in our country, and justly ranks with the first medical men of the age. The following sketch of his medical career, will fully sustain his claims to the high position which he occupies.

"Dr. Bell pursued his preliminary medical studies in Va., and when just of age, received the Degree of Doctor of Medicine from the University of Pennsylvania, after he had attended the regular lectures required for the occasion. He went abroad immediately afterwards, and was absent four years: part of the time as a surgeon in vessels in the China trade, a part in attending the hospitals and lectures on practical subjects in France and Italy, and for a short period in England," &c.

The Announcement of Jefferson Medical College has the following:—

"After several years of great prosperity, the catalogue of students during the last session attained the number, unprecedented in the history of this or of any other institution in the country, of *five hundred and sixteen;* whilst that of the graduates was *two hundred and eleven.* So ready—as the Faculty have in former years remarked—is now the communication between the most distant portions of this wide-spread country, and so generally established is the position, that the great principles of medical science are the same everywhere, that the student, untrammelled by sectional prejudices, unhesitatingly goes in search of information wherever it is admitted that it can be best obtained.

In evidence of the prevalent and increasing feeling among the profession, that it is desirable for the student to visit Philadelphia for at least one session, it may be sufficient to glance at the number from other institutions who formed part of the class of the Jefferson Medical College during the last session. Of the five hundred and sixteen, *one hundred and sixty-two* were in this category."

The Circular of the University of St. Louis, says:—

"Indeed, considering the superior advantages and position of St. Louis, as compared with all other places in the West, its easy access from every direction and at all seasons, its unexampled increase in commerce and population, its extensive and well-filled hospitals, and abundance of Anatomical Material, few can deny that it is destined rapidly to become the seat of a great Medical School Fully alive to these advantages, the Faculty are resolved to turn them to good account, and to spare no effort, which will advance the best interests of medical education, extend the usefulness of their Institution, and make St. Louis the medical, as she already is the commercial metropolis of the Great West. Linked as the school is with the rise and progress of a great and flourishing city, their mutual destiny is to a high and useful future."

The Announcement of the College of Physicians and Surgeons of New York city (old school), discourses as follows:—

"It is time, for the credit of our common country, that the necessity of visiting foreign schools and hospitals to complete a medical education should be done away with, and that our medical instructions should take their proper place, not as introductions to, or preparatives for, European education, but as, of themselves, and in themselves, complete and independent. Such a position, the Trustees of the College of Physicians and Surgeons have ever desired and designed to secure for the institution committed to their care, and to such a position they now believe it entitled, by its situation in the commercial metropolis of the country, by its connection with both the great hospitals of New York, in each of which its Professors have abundant opportunities of giving clinical instruction, and by the zeal and industry with which the Faculty strive to improve to the utmost their great advantages." E.

